# Regional Expression of Vimentin, S100, and Epithelial Membrane Antigen in the Human Medial Collateral Ligament: A Robust Two-Way Analysis of Variance

**DOI:** 10.3390/jfmk11020173

**Published:** 2026-04-25

**Authors:** Nikola Stamenov, Boycho Landzhov, Maria Piagkou, Ahmed Al-Sadek, Lyubomir Gaydarski, Kristina Petrova, Georgi Luchev, Julian Ananiev, Iva N. Dimitrova, Georgi P. Georgiev

**Affiliations:** 1 Department of Anatomy, Histology and Embryology, Medical University of Sofia, 1431 Sofia, Bulgaria; nstamenov@medfac.mu-sofia.bg (N.S.); blandzhov@medfac.mu-sofia.bg (B.L.); lgaydarski@medfac.mu-sofia.bg (L.G.); kpetrova@medfac.mu-sofia.bg (K.P.); 2 Department of Anatomy, School of Medicine, National and Kapodistrian University of Athens, 11527 Athens, Greece; piagkoumara@gmail.com; 3 Department of Orthopedics and Traumatology, University Hospital Queen Giovanna-ISUL, Medical University of Sofia, 1527 Sofia, Bulgaria; aalsadek@medfac.mu-sofia.bg (A.A.-S.); georgi.luchev06@gmail.com (G.L.); 4 Department of General and Clinical Pathology, Faculty of Medicine, Trakia University, 6000 Stara Zagora, Bulgaria; operation@abv.bg; 5 Department of Cardiology, University Hospital “St. Ekaterina”, Medical University of Sofia, 1431 Sofia, Bulgaria; dimytrova@yahoo.com

**Keywords:** epiligament, ligament proper, ligament healing, immunohistochemistry, fibroblasts, vascularization, neural components, knee ligament, connective tissue healing

## Abstract

**Background**: The epiligament (EL) of the medial collateral ligament (MCL) has recently attracted increasing attention as a biologically active structure. Emerging evidence suggests that it may contribute to ligament healing by providing progenitor cells, vascular components, and signaling mediators. However, its cellular composition and possible regional variability remain insufficiently characterized. **Aim**: This study evaluated the expression of vimentin, S100 protein, and epithelial membrane antigen (EMA) to better characterize the EL compared with the ligament proper (LP). **Methods**: Twelve human MCLs obtained from twelve deceased donors were divided into proximal, middle, and distal segments. Thirty-six paraffin blocks were prepared, from which 180 sections were obtained and equally assigned for immunohistochemical staining of vimentin, S100 protein, and EMA (60 slides for each marker). Systematic quantification of seven to eight non-overlapping microscopic fields per slide generated 900 standardized observations for each investigated marker. This sampling strategy provided 150 measurements for each sub-region (EL and LP across the three anatomical segments). Immunoreactivity was quantified using ImageJ software. Statistical differences were analyzed using a robust two-way analysis of variance (ANOVA), while biological associations between markers were assessed using Spearman’s rank correlation analysis. **Results**: Vimentin and S100 expression were consistently higher in the EL than in the LP across all anatomical regions (*p* < 0.0001). The highest vimentin values were observed in the proximal region (median 17.34 vs. 10.14) and distal region (median 19.34 vs. 11.23), whereas S100 showed the greatest expression in the proximal (median 16.9 vs. 7.2) and distal regions (median 14.1 vs. 8.9). EMA expression was generally lower overall; however, it remained significantly higher in the EL than in the LP within the proximal (median 6.87 vs. 5.77) and middle regions (median 4.80 vs. 3.26). No significant difference was identified in the distal region. Spearman rank correlation analysis demonstrated significant positive associations among all investigated markers (*p* < 0.001), with the strongest relationship observed between vimentin and S100 protein (Spearman correlation coefficient = 0.430). **Conclusions**: The EL of the MCL is a structurally and biologically distinct component, characterized by significantly higher expressions of vimentin, S100, and EMA than the LP. The significant positive correlations observed among these markers support the concept that the EL functions as an integrated biological microenvironment with clear regional heterogeneity, particularly within the proximal and distal segments. Further studies are warranted to clarify the functional relevance of these findings and their potential implications for clinical management and ligament healing strategies.

## 1. Introduction

The medial collateral ligament (MCL) is a broad and robust structure situated along the medial aspect of the knee joint. It consists of two principal components: the superficial medial collateral ligament (sMCL) and the deep medial collateral ligament (dMCL) [1,2]. Functionally, the MCL plays a critical role in resisting valgus stress and stabilizing the knee during external rotation [2].

The MCL is the most frequently injured ligament in the knee, accounting for approximately 90% of all knee ligament injuries. This high clinical prevalence has generated considerable research interest and an extensive body of literature focused on its distinctive biological and regenerative properties [3]. MCL injuries commonly result from valgus loading during athletic activities such as football, ice hockey, and skiing [4]. Reported incidence rates range from 0.24 to 7.3 cases per 1000 persons, indicating that MCL injury is common in the general population and occurs approximately twice as often in men as in women [5,6]. In contrast, isolated injury to the lateral collateral ligament (LCL) is uncommon, representing only about 2% of ligamentous knee injuries. Whereas the MCL is frequently injured in isolation, LCL trauma usually occurs in association with injuries to other posterolateral corner structures [7].

Surrounding the MCL is a thin and less dense connective tissue layer known as the epiligament (EL) [3,8]. The EL is regarded as a distinct structural compartment characterized by greater cellularity and a richer vascular network than the ligament proper (LP) [8,9]. It contains a heterogeneous population of cells, including fibroblasts, fibrocytes, adipocytes, endothelial cells, mast cells, and neural elements such as mechanoreceptors [10,11]. Previous studies have suggested that the EL may contribute to ligament homeostasis and healing; however, its precise biological role remains incompletely understood [3,12].

Given the high frequency of MCL injuries and their relatively favorable healing potential compared with intra-articular ligaments, a better understanding of the EL is of clear clinical importance [12,13]. We hypothesized that the EL would demonstrate a distinct biochemical profile compared with the LP, reflecting their different structural characteristics. We further hypothesized that marker expression would vary significantly across the proximal, middle, and distal regions of the ligament. Such findings could provide deeper insight into the biological heterogeneity of the MCL and may support future advances in both conservative and surgical treatment strategies.

Vimentin is a type III intermediate filament protein (57 kDa) that forms an important part of the cytoskeleton in mesenchymal cells [14,15,16]. It is expressed in several cell types, including fibroblasts, endothelial cells, chondroblasts, leukocytes, and Schwann cells [15]. Beyond providing structural support, vimentin is involved in key cellular processes, including migration, proliferation, and mechanotransduction, all of which are essential for tissue repair [15,17].

The S100 protein family comprises more than 20 small calcium-binding proteins, typically ranging from 9 to 14 kDa [18,19]. Upon binding calcium, these proteins undergo conformational changes that enable interactions with multiple intracellular targets [20]. They regulate processes including cell adhesion, motility, proliferation, and apoptosis. In addition, certain S100 proteins function extracellularly as signaling molecules involved in inflammation and tissue repair [18,21].

Epithelial membrane antigen (EMA), also known as mucin 1 (MUC1), is a transmembrane glycoprotein involved in barrier formation, lubrication, and modulation of inflammatory responses [22,23,24,25]. Although primarily expressed in epithelial tissues, EMA is also expressed in selected non-epithelial cells, including fibroblasts, where it may influence cell adhesion and migration [26].

Despite increasing interest in EL, limited information is available regarding the regional distribution of fibroblastic, neural-related, and vascular components within this tissue. Therefore, the present study evaluated the immunohistochemical expression of vimentin, S100, and EMA in both the EL and LP of the human MCL. By comparing these markers across anatomical regions, this study sought to provide a clearer understanding of the structural and biological organization of EL.

## 2. Materials and Methods

This study was conducted as an anatomical and immunohistochemical investigation of the human MCL at the Department of Anatomy, Histology and Embryology, Medical University of Sofia, Bulgaria. The research was performed in accordance with the Declaration of Helsinki and received formal approval from the Medico-Legal Office and the Local Ethics Committee (Approval No. 16/1 August 2024). As the study used cadaveric material provided for scientific purposes under institutional regulations, informed consent from family members was not applicable. The investigation was based on the premise that the EL represents a structurally and biologically distinct layer when compared with the LP [8,27].

### 2.1. Specimen Collection

A total of twelve fresh human cadaveric knee specimens obtained from 12 donors with intact MCLs were included in the study. The mean donor age was 54 years, with equal representation of male and female individuals. Specimens were excluded if there was evidence of previous ligament injury, prior surgical intervention, advanced medial compartment osteoarthritis, infection, neoplastic disease, or significant postmortem tissue deterioration.

All ligaments were collected in strict accordance with the standardized protocol described by Kholinne et al. [28]. Each MCL was carefully exposed and dissected, with particular attention given to preserving the surrounding EL tissue. For regional analysis, each ligament was divided into proximal, middle, and distal segments. This subdivision was selected based on previous findings indicating that ligament structure and vascular supply vary along its longitudinal axis [8,9].

### 2.2. Tissue Preparation

Following dissection, all specimens were fixed in 10% neutral buffered formalin. Standard histological processing was subsequently performed, including dehydration through graded alcohol solutions, clearing in xylene, and paraffin embedding.

A total of thirty-six paraffin blocks were prepared, representing the three anatomical regions (proximal, middle, and distal) of the twelve MCLs. From each block, five representative sections were obtained at a minimum interval of 100 µm to ensure systematic and objective sampling throughout the tissue volume, resulting in a total of 180 slides. Sections were cut at 6 µm using a rotary microtome and mounted on coated glass slides.

The 180 slides were equally allocated among the three investigated markers—vimentin, S100 protein, and EMA—yielding sixty sections for each marker. For every section, seven to eight non-overlapping microscopic fields were randomly selected and quantified using ImageJ 1.54g software. This sampling strategy generated 900 standardized observations per marker and was structured to provide 150 data points for the EL and 150 for the LP within each anatomical region. This systematic approach provided a representative dataset for robust two-way analysis of variance and Spearman rank correlation analyses.

Each anatomical region was processed separately to preserve orientation and enable accurate regional comparisons. Particular care was taken throughout the procedure to maintain the interface between the EL and the LP.

### 2.3. Immunohistochemistry

Immunohistochemical staining for vimentin, S100, and EMA was performed using a standardized protocol. Tissue sections (6 µm thick) were first deparaffinized in xylene, rehydrated through a graded series of decreasing alcohol concentrations, and rinsed in distilled water.

Antigen retrieval was performed by heating the sections at 95 °C for 20 min in a citrate buffer (pH 6.0; ScyTek Laboratories Inc., Logan, UT, USA). After cooling, the slides were washed in Tween-buffered Tris-saline (TTBS). Endogenous peroxidase activity was blocked with 3% hydrogen peroxide for 10 min.

To reduce non-specific staining, sequential blocking procedures were applied, including Super Block, an endogenous biotin-blocking system, and a mouse-to-mouse blocking kit, all used according to the manufacturers’ instructions. Sections were then incubated overnight at 4 °C with the following primary antibodies:

- S100 protein (polyclonal rabbit, ready-to-use, Dako, Glostrup, Denmark);
- Vimentin (monoclonal mouse, Clone 9, ready-to-use, Dako);
- EMA (mouse monoclonal, dilution 1:100, Leica Biosystems, Nussloch, Germany).

Signal detection was performed using a horseradish peroxidase (HRP)-based system with 3,3’-diaminobenzidine (DAB) as the chromogen. Slides were counterstained with Mayer’s hematoxylin, dehydrated, cleared, and coverslipped. Negative controls were processed in parallel by omission of the primary antibody.

Comparisons were performed between the EL and LP for each specimen across all three anatomical regions (proximal, middle, and distal).

### 2.4. Immunohistochemical Assessment

Staining intensity was quantified using ImageJ software with the IHC ToolBox 1.0.0 (2014) plugin. Mean gray values were inverted before analysis. All images were acquired under identical imaging conditions, and the same threshold parameters were applied throughout to ensure methodological consistency.

From each of the thirty-six paraffin blocks (12 specimens × 3 anatomical regions), five representative sections were selected. From every section, seven to eight non-overlapping microscopic fields were captured and analyzed, yielding 150 measurements for each sub-region and investigated marker. This resulted in a total of 900 observations across the study. Regions of interest were manually defined, and all measurements were performed by an observer blinded to sample identity. All sections were stained in a single batch and imaged using fixed acquisition settings, thereby minimizing technical variability.

### 2.5. Statistical Analysis

Each marker was analyzed independently. Data normality was assessed using the Shapiro–Wilk test, while homogeneity of variances was evaluated using Levene’s test. Differences between groups were examined using two-way analysis of variance (ANOVA), with tissue type (EL vs. LP) and anatomical region (proximal, middle, and distal) entered as fixed factors. Because heterogeneity of variance was identified, a robust HC3-adjusted ANOVA model was applied. Significant overall effects were followed by pairwise comparisons using Welch’s *t*-test with Holm correction for multiple testing. Effect sizes were reported as partial eta squared (η^2^) for ANOVA and Cohen’s d for pairwise comparisons. Results are presented as median and interquartile range (IQR) to better reflect the biological variability of the human specimens. Statistical significance was defined as *p* < 0.05. All analyses were performed in Python 3.14 using SciPy 1.17.1, statsmodels, and pandas libraries, following procedures comparable to those used in SPSS 31.0.2.0. For visual clarity, results for each tissue type are presented in separate panels, while the statistical model accounts for all factors simultaneously. Although individual donor identifiers were not available, observations were treated as independent for statistical purposes. The low variability of the data and the use of standardized staining conditions suggest that the observed differences were predominantly biological rather than technical in origin. Spearman’s rank correlation analysis was performed to examine relationships among the three investigated markers. This non-parametric approach was selected because of the non-normal distribution and heterogeneity of variance observed in the quantitative immunohistochemical data. The analysis was based on 900 standardized observations per marker, allowing identification of global biological trends and coordinated expression patterns across the investigated structures. The strength of monotonic associations between marker pairs was expressed using Spearman’s rho (r_s_). All tests were two-tailed, and statistical significance was set at *p* < 0.05. All procedures related to tissue processing, immunohistochemical staining, and quantitative assessment were conducted in strict accordance with the established protocols previously described by Georgiev et al. [3,27].

## 3. Results

Histological evaluation of the human MCL revealed clear, consistent structural differences between the EL and the LP. The EL formed the outermost layer and exhibited looser connective tissue organization, increased cellularity, and more heterogeneous morphology. Blood vessels were readily identifiable within this region. In contrast, the LP displayed the typical characteristics of dense regular connective tissue, including tightly packed collagen fibers, lower cellular density, and limited vascularization. These differences were consistently observed across all specimens and anatomical regions—proximal, middle, and distal—allowing reliable distinction between the two structural compartments.

### 3.1. Vimentin Expression

**Figure 1:**
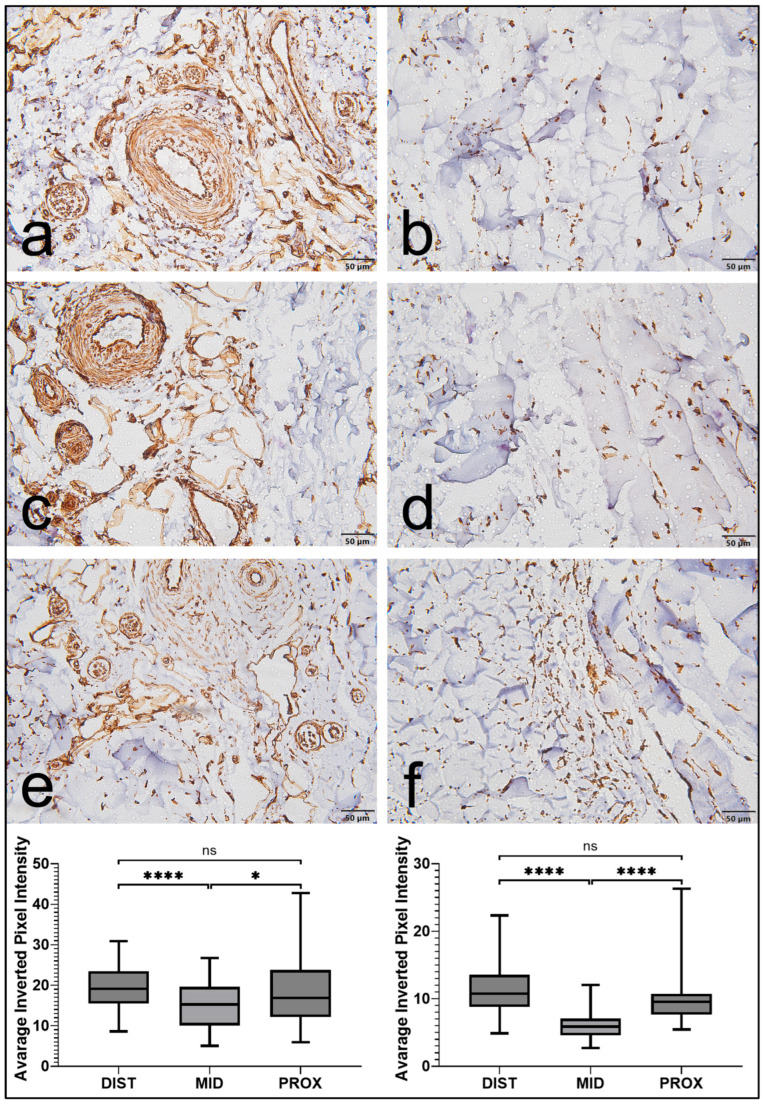
Vimentin expression in the epiligament (EL) and ligament proper (LP) of the human medial collateral ligament (MCL). (**a**,**c**,**e**) Representative micrographs showing vimentin immunoreactivity in the EL across proximal, middle, and distal regions, demonstrating strong staining in fibroblast-like and vascular-associated cells. (**b**,**d**,**f**) Corresponding sections of the LP showed weaker and more limited staining. Scale bars: 50 μm. Regional variation in staining intensity in the MCL. Box plots represent quantitative analysis of staining intensity in the distal, middle, and proximal regions of the epiligament (EL, left) and ligament proper (LP, right). In both tissue types, the middle region showed significantly lower expression than the proximal and distal regions, whereas no significant difference was observed between the proximal and distal regions. Data are presented as the median and interquartile range, with whiskers indicating the minimum and maximum values. Statistical analysis was performed using Welch’s *t*-test with Holm correction. * *p* < 0.05, **** *p* < 0.0001, ns = not significant.

Vimentin immunostaining was detected in both the EL and the LP; however, staining was consistently more extensive and intense in the EL (Figure 1). Within the EL, immunoreactivity was primarily observed in elongated spindle-shaped cells morphologically consistent with fibroblasts, as well as in cells associated with vascular structures. In contrast, vimentin expression in the LP was weaker and more localized.

Quantitative analysis using two-way ANOVA demonstrated significant main effects of tissue type (F(1, 894) = 793.84, *p* < 0.0001) and anatomical region (F(2, 894) = 199.69, *p* < 0.0001) on vimentin expression. No significant interaction between tissue type and region was observed (*p* = 0.69), indicating that the difference between the EL and LP remained consistent along the entire length of the ligament.

**Figure 2:**
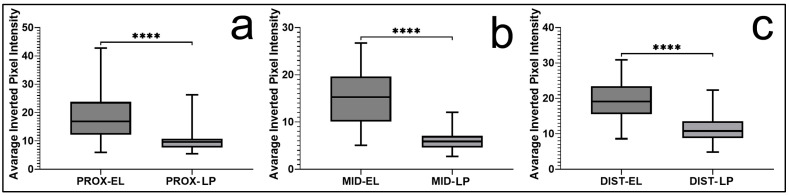
Regional comparison of vimentin expression between the epiligament (EL) and ligament proper (LP). Box plots represent quantitative analysis of staining intensity in the proximal (**a**), middle (**b**), and distal (**c**) regions of the medial collateral ligament (MCL). In all regions, expression was significantly higher in the epiligament (EL) compared to the ligament proper (LP). Data are presented as the median and interquartile range, with whiskers indicating the minimum and maximum values. Statistical analysis was performed using Welch’s *t*-test with Holm correction. **** *p* < 0.0001.

Direct comparisons further confirmed that vimentin expression was significantly higher in the EL than in the LP across all regions (*p* < 0.0001; Figure 2). When regional variation was examined within both tissue compartments, expression was greater in the proximal and distal regions than in the middle portion. The middle region demonstrated significantly lower values (*p* < 0.05). In contrast, no significant difference was identified between the proximal and distal segments (Figure 1). Overall, vimentin staining in the EL was more extensive and followed a consistent regional biological pattern throughout the ligament.

### 3.2. S100 Expression

Immunohistochemical evaluation demonstrated that the S100 protein was present in both the EL and the LP, although its distribution was clearly more extensive in the EL. Within this outer layer, S100 positivity was primarily observed in cells morphologically resembling Schwann cells or other neural-associated elements, as well as in selected fibroblast-like cells and perivascular structures. In contrast, the LP exhibited weaker staining with a more limited and less uniform distribution.

**Figure 3:**
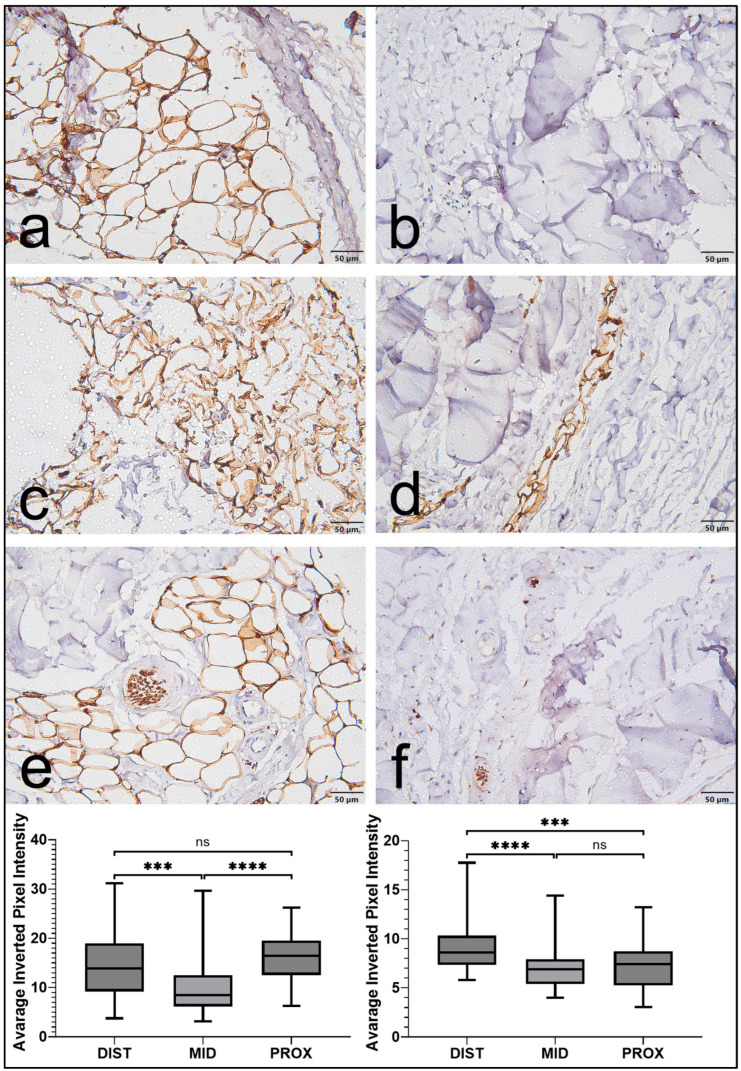
S100 expression in the epiligament (EL) and ligament proper (LP) of the human medial collateral ligament. (**a**,**c**,**e**) Representative micrographs of the epiligament showing strong S100 immunoreactivity in neural-associated and perivascular structures across proximal, middle, and distal regions. (**b**,**d**,**f**) Corresponding sections of the LP show weaker, less uniform staining. Scale bars: 50 μm. Quantitative analysis of staining intensity is presented as box plots for the EL (left) and LP (right) across distal (DIST), middle (MID), and proximal (PROX) regions. Statistical analysis was performed using two-way ANOVA followed by Welch’s *t*-test with Holm correction. Significance levels: *** *p* < 0.001, **** *p* < 0.0001, ns = not significant.

Statistical analysis using two-way ANOVA revealed significant main effects for tissue type (F(1, 894) = 505.19, *p* < 0.0001) and anatomical region (F(2, 894) = 47.68, *p* < 0.0001). A significant interaction between these factors was also identified (F(2, 894) = 40.24, *p* < 0.0001), indicating that the difference in S100 expression between the EL and LP varied across regions. Subsequent pairwise comparisons confirmed that S100 expression was significantly higher in the EL than in the LP in all anatomical regions (*p* < 0.0001), with the greatest differences observed in the proximal (median 16.9 vs. 7.2) and distal (median 14.1 vs. 8.9) segments of the ligament (Figure 3).

**Figure 4:**
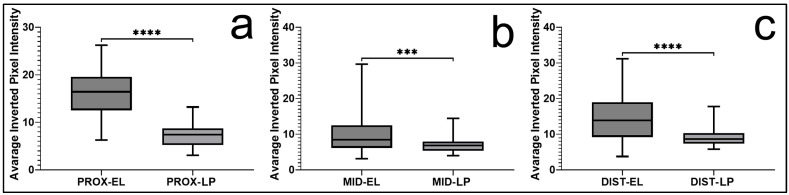
Regional comparison of S100 expression between the epiligament (EL) and ligament proper (LP). Box plots represent quantitative analysis of staining intensity in the proximal (**a**), middle (**b**), and distal (**c**) regions of the medial collateral ligament. S100 expression was significantly higher in the EL compared to the LP in all regions. Data are presented as the median and interquartile range, with whiskers indicating the minimum and maximum values. Statistical analysis was performed using Welch’s *t*-test with Holm correction. *** *p* < 0.001, **** *p* < 0.0001.

When regional variation within the EL was assessed, S100 expression was significantly higher in both the proximal and distal regions than in the middle portion. The middle region (median 9.4) demonstrated significantly lower values than the proximal (*p* < 0.0001) and distal (*p* < 0.001) regions, whereas no significant difference was observed between the proximal and distal segments. Overall, S100 staining followed a consistent biological pattern, characterized by stronger and more widespread expression in the EL together with clear regional heterogeneity along the length of the MCL (Figure 4).

### 3.3. EMA Expression

Immunohistochemical staining confirmed the presence of EMA in the human MCL. Compared with vimentin and S100, EMA staining was generally less intense. In both the EL and the LP, EMA immunoreactivity was predominantly localized around vascular and perivascular structures.

**Figure 5:**
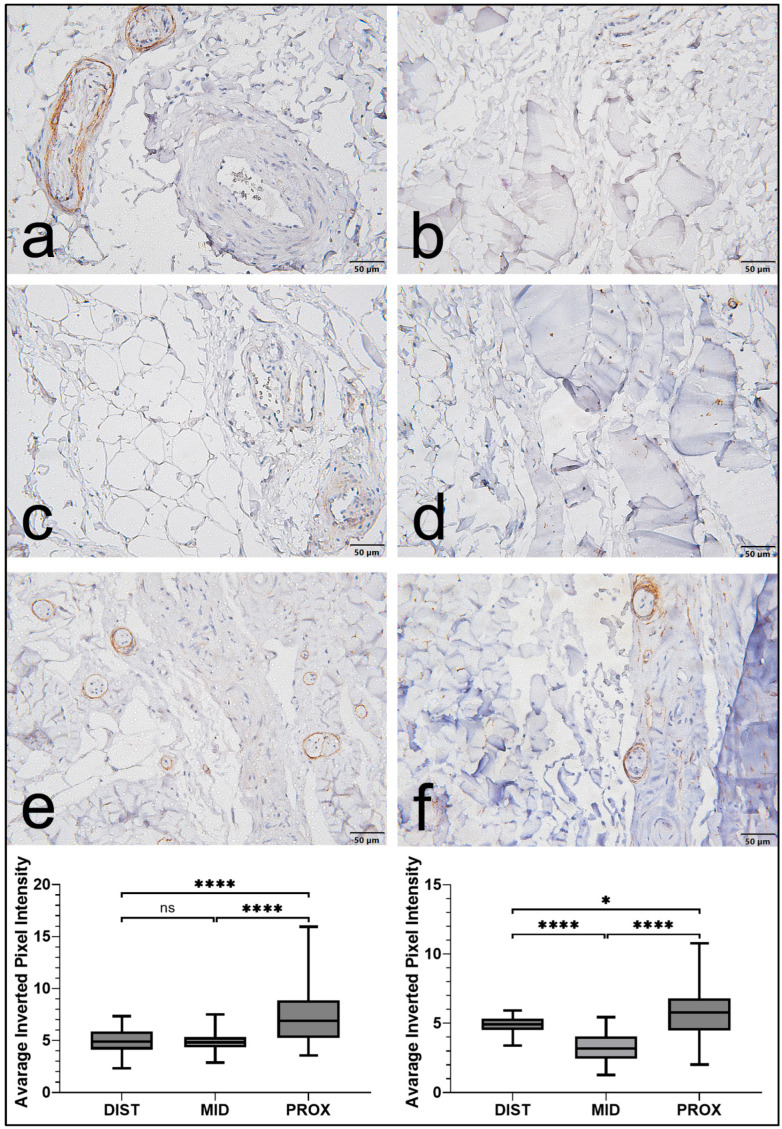
EMA expression in the epiligament (EL) and ligament proper (LP) of the human medial collateral ligament. (**a**,**c**,**e**) Representative micrographs of the epiligament showing EMA immunoreactivity primarily localized to vascular and perivascular structures across proximal, middle, and distal regions. (**b**,**d**,**f**) Corresponding sections of the LP show weaker, more limited staining. Scale bars: 50 μm. Quantitative analysis of staining intensity is presented as box plots for the epiligament (left) and ligament proper (right) across distal (DIST), middle (MID), and proximal (PROX) regions. Statistical analysis was performed using two-way ANOVA followed by Welch’s *t*-test with Holm correction. * *p* < 0.05, **** *p* < 0.0001, ns = not significant.

Statistical analysis using two-way ANOVA demonstrated significant main effects for both tissue type (F(1, 894) = 140.03, *p* < 0.0001) and anatomical region (F(2, 894) = 204.24, *p* < 0.0001). A significant interaction between these factors was also observed (F(2, 894) = 45.45, *p* < 0.0001), indicating that differences in EMA expression between the EL and LP varied significantly across anatomical regions. When regional patterns were examined, EMA expression was highest in the proximal region and gradually decreased toward the middle and distal segments. The middle region (median 3.26 in LP) showed significantly lower expression than the proximal region (*p* < 0.0001), whereas no significant difference was identified between the middle and distal regions (Figure 5).

**Figure 6:**
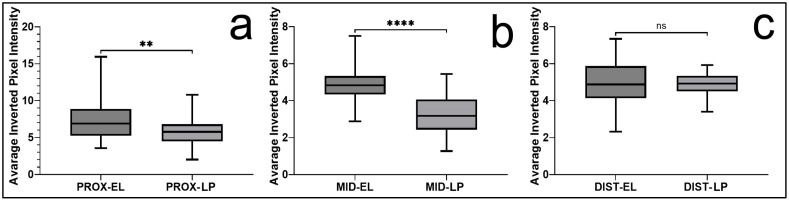
Regional comparison of EMA expression between the epiligament (EL) and ligament proper (LP). Box plots represent quantitative analysis of staining intensity in the proximal (**a**), middle (**b**), and distal (**c**) regions of the medial collateral ligament (MCL). EMA expression was significantly higher in the EL compared to the LP in the proximal and middle regions, while no significant difference was observed distally. Data are presented as the median and interquartile range, with whiskers indicating the minimum and maximum values. Statistical analysis was performed using Welch’s *t*-test with Holm correction. ** *p* < 0.01, **** *p* < 0.0001, ns = not significant.

Direct comparisons between tissue compartments revealed that EMA expression was significantly higher in the EL than in the LP in both the proximal (median 6.87 vs. 5.77, *p* < 0.01) and middle (median 4.80 vs. 3.26, *p* < 0.0001) regions. In the distal region, however, the difference between the two tissues was not statistically significant (median 4.86 vs. 4.93, *p* > 0.05). Overall, EMA staining appeared closely associated with vascular elements and demonstrated a clear region-dependent biological pattern along the length of the ligament (Figure 6).

### 3.4. Correlation Analysis

To evaluate biological coordination among cellular, neural, and vascular components within the human MCL, Spearman’s rank correlation analyses were performed to assess associations between vimentin, S100, and EMA expression across all sub-regions. Vimentin expression demonstrated the strongest and most consistent positive correlation with S100. A significant positive association was identified between these markers (r_s_ = 0.430, *p* < 0.001), suggesting that fibroblast-like cell density is closely related to the presence of neural-associated structures in both the EL and the LP.

Vimentin also showed a significant positive correlation with EMA (r_s_ = 0.312, *p* < 0.001), indicating that increased mesenchymal cellularity was associated with greater vascular density along the length of the ligament. In addition, a significant positive association was observed between S100 and EMA expression (r_s_ = 0.244, *p* < 0.001), supporting a spatial relationship between neuroregulatory elements and the vascular network.

**Figure 7:**
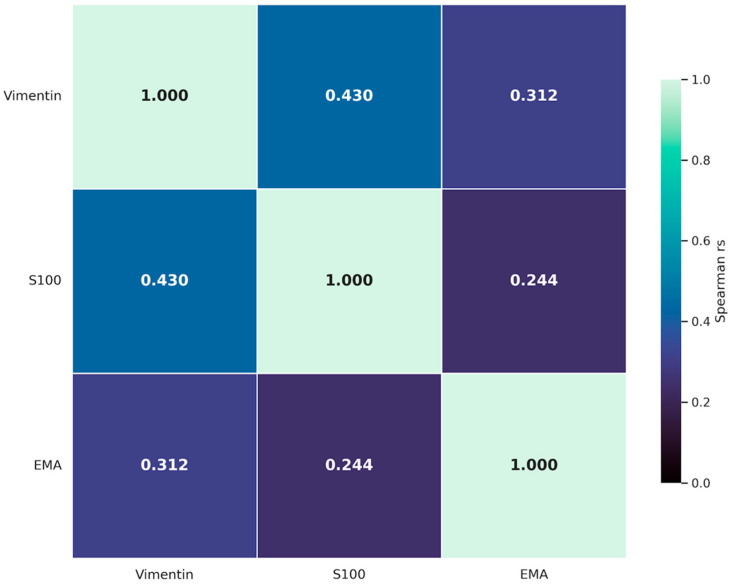
Heatmap of the Spearman correlations between vimentin, S100, and EMA expression in the human MCL. The heatmap displays Spearman rank correlation coefficients (r_s_) for each marker across all sub-regions, based on 900 individual observations (150 per sub-region). All markers demonstrate statistically significant positive correlations (*p* < 0.001), indicating a spatially synchronized biological microenvironment where fibroblastic, neural, and vascular components are strategically coordinated to support tissue maintenance and repair. Color intensity represents the strength of the positive correlation (r_s_), with the strongest association observed between vimentin and S100 (r_s_ = 0.430).

Collectively, these findings suggest that vimentin is a central correlate within the MCL microenvironment, while the significant positive relationships among all three markers support the presence of an integrated biological system in which fibroblastic, neural, and vascular components are coordinately organized to support tissue maintenance and repair. A summary of the correlation analysis is presented in Figure 7.

## 4. Discussion

The primary objective of this investigation was to characterize the cellular, neural, and vascular organization of the human MCL through evaluation of vimentin, S100, and EMA expression. Specifically, the study aimed to quantify biological differences between the EL and the LP, and to determine whether these markers exhibited significant regional variation across the proximal, middle, and distal segments of the tissue. By comparing these two distinct structural compartments, we sought to provide a clearer understanding of the internal biological microenvironment of the MCL and its potential contribution to tissue maintenance and repair.

### 4.1. Vimentin Expression and Fibroblast Distribution

The present findings demonstrate that vimentin expression was consistently higher in the proximal and distal regions of the EL than in the LP, with the greatest relative difference observed in the middle region. This pattern likely reflects the greater cellularity of the EL, which contains more fibroblasts and other mesenchymal-derived cells than the more sparsely populated LP [29]. These observations agree with the current understanding of ligament healing, in which fibroblasts play a central role in extracellular matrix synthesis and tissue remodeling [13,30]. It should be emphasized, however, that the present results are descriptive in nature and do not directly establish functional mechanisms.

The contribution of vascular-associated cells should also be considered. EL is known to possess a richer vascular network, which may partly explain the stronger vimentin staining observed in this layer [8]. Vascularization is essential for ligament repair, as it supports nutrient delivery, cell migration, and overall metabolic activity [9,12].

Structural differences between the EL and LP may further influence this distribution. While the LP specializes primarily in mechanical strength, EL has a looser and more adaptable organization that likely facilitates cellular movement. In this context, vimentin may be associated with cytoskeletal support and with cells’ ability to respond to mechanical stress [17]. The increased expression observed in the proximal and distal regions may also reflect enhanced mechanical and biological activity at the ligament insertions (entheses), which are recognized as functionally dynamic zones [31,32].

Previous studies have identified progenitor cells and myofibroblasts within the EL, particularly near these insertion sites [11]. This localized enrichment supports the concept that regions exposed to greater mechanical demands may also harbor more active populations of reparative cells [33].

### 4.2. Neural Components and S100 Expression

A similar distribution pattern was observed for the S100 protein, with consistently higher expression in the EL than in the LP across all anatomical regions. Positive staining was primarily identified in cells with neural-like characteristics, as well as in perivascular and fibroblast-like elements. These findings are consistent with previous studies reporting S100 expressions in Schwann cells, mechanoreceptors, and selected fibroblast populations involved in tissue repair [18].

Beyond structural support, neural elements are now recognized as active regulators of tissue healing. Peripheral nerves release neuropeptides such as substance P and calcitonin gene-related peptide (CGRP), both of which may influence inflammation, angiogenesis, and fibroblast activity [10]. In this context, the increased S100 expression observed in the EL may reflect a more developed neuroregulatory microenvironment within this layer.

### 4.3. Vascular Features and EMA Expression

EMA demonstrated a different distribution pattern compared with vimentin and S100. Although overall expression was generally higher in the EL, the differences were less pronounced and varied across anatomical regions. The most notable differences were observed in the proximal and middle regions, whereas no significant distinction between the EL and LP was identified in the distal region.

EMA staining was predominantly localized to vascular and perivascular structures, consistent with its known tissue distribution [26]. The regional variation observed in the present study may reflect differences in vascular organization along the length of the ligament. Previous studies have suggested that vascular density is not uniform and may be greater near ligament insertions, potentially influencing local biological activity and healing capacity [9].

### 4.4. Integrated Microenvironment of the Epiligament

Taken together, the expression patterns of vimentin, S100, and EMA highlight the complex cellular composition of the EL. The coexistence of fibroblastic, neural-related, and vascular elements suggests an integrated microenvironment within this structure. Although the present study does not directly investigate functional interactions, the spatial association of these components supports the concept of coordinated biological activity within the EL.

These findings are consistent with broader principles of connective tissue biology, which emphasize the interplay between cellular, vascular, and neural systems in maintaining tissue integrity and supporting repair processes [12,33]. Within this framework, EL may serve as an important interface contributing to both structural support and biological responsiveness.

A key finding of the present study is the significant positive correlation among fibroblastic (vimentin), neural (S100), and vascular (EMA) markers, providing quantitative support for the concept of an integrated microenvironment within the MCL epiligament [8]. The strongest association was observed between vimentin and S100 (r_s_ = 0.430, *p* < 0.001), suggesting spatial and functional coordination between mesenchymal reparative cells and the neuroregulatory apparatus, including mechanoreceptors [11]. This relationship is biologically relevant, as neural elements within ligaments have been shown to release neuropeptides such as substance P and calcitonin gene-related peptide (CGRP), which may directly influence fibroblast proliferation and extracellular matrix remodeling [10].

Furthermore, the positive correlation between vimentin and EMA (r_s_ = 0.312, *p* < 0.001) indicates that regions of increased mesenchymal cellularity are closely associated with a richer vascular supply [9]. The relationship between S100 and EMA (r_s_ = 0.244, *p* < 0.001) further suggests the presence of organized neurovascular units within the EL [8]. Unlike the LP, which is specialized mainly for mechanical strength and resistance to valgus stress, the EL appears to function as a biologically specialized compartment [12]. These findings support the clinical rationale for preserving the EL during surgical procedures in order to maintain this integrated biological machinery, which may contribute to the superior healing capacity of the MCL compared with the ACL [26,29].

### 4.5. Functional and Clinical Implications

The structural and cellular organization of the EL may have important implications for ligament healing and functional recovery. The presence of fibroblastic, vascular, and neural components suggests that this layer may contribute not only to reparative processes but also to proprioception and adaptation to mechanical loading. From a clinical perspective, these observations support surgical strategies that preserve EL whenever feasible. In addition, the EL may represent a promising target for future biological therapies. However, such potential applications remain hypothetical and require further experimental and translational validation.

### 4.6. Limitations and Future Directions

Several limitations of the present study should be acknowledged. First, the investigation was based on immunohistochemical analysis and was therefore primarily descriptive. Although this approach allows identification of regional differences in protein expression, it does not provide direct evidence of functional activity or causal mechanisms. Second, the relatively modest sample size may limit the generalizability of the findings. In addition, all specimens were obtained from non-injured cadaveric tissue, which may not fully represent the biological conditions present during acute injury or active healing. Third, while the results support the hypothesis that the EL functions as a specialized biological compartment, future studies should move beyond morphological quantification toward direct functional analyses. Finally, only a limited number of markers were examined. Other relevant pathways, including angiogenesis, extracellular matrix remodeling, inflammation, and progenitor cell activity, were not directly assessed.

Future investigations should incorporate molecular techniques, biomechanical testing, and experimental injury models to further define the role of the EL in ligament repair and determine whether these expression patterns are maintained under pathological conditions.

## 5. Conclusions

This investigation provides strong evidence that the EL of the MC is a structurally and biologically distinct component, characterized by increased cellularity, vascularization, and a greater presence of neural-associated elements compared with the LP. The elevated expression of vimentin, S100, and EMA observed in the present study reflects a complex and highly integrated biological microenvironment. Furthermore, the significant positive correlations among these markers suggest that fibroblastic, neural, and vascular components are coordinately organized to support cellular migration, tissue maintenance, and structural adaptation.

The study also demonstrates clear regional heterogeneity, identifying proximal and distal segments as the principal zones of biological activity. From a translational perspective, preservation of the EL during surgical procedures may help maintain this integrated biological machinery, which could contribute to the superior healing capacity of the MCL compared with the ACL. In addition, the EL may represent a promising target for future biological therapies. Although these immunohistochemical findings do not establish direct functional mechanisms, they provide a robust foundation for future experimental and clinical studies to clarify the precise role of EL in ligament healing and its therapeutic potential.

## Data Availability

The original contributions presented in this study are included in the article. Further inquiries can be directed to the corresponding author.

## Abbreviations

The following abbreviations are used in this manuscript:

MCL	Medial collateral ligament
EL	Epiligament
LP	Ligament proper
EMA	Epithelial membrane antigen
MUC1	Mucin 1
sMCL	Superficial medial collateral ligament
dMCL	Deep medial collateral ligament
ANOVA	Analysis of variance
kDa	Kilodalton
µm	Micrometer
TTBS	Tween-buffered Tris-saline
HRP	Horseradish peroxidase
DAB	3,3’-Diaminobenzidine
CGRP	Calcitonin gene-related peptide
LCL	Lateral collateral ligament

## References

[1] Robinson J.R., Bull A.M., Amis A.A. (2005). Structural properties of the medial collateral ligament complex of the human knee. J. Biomech..

[2] Ball S., Stephen J., El-Daou H., Williams A., Amis A.A. (2020). The medial ligaments and the ACL restrain anteromedial laxity of the knee. Knee Surg. Sports Traumatol. Arthrosc..

[3] Georgiev G.P., Yordanov Y., Gaydarski L., Tubbs R.S., Olewnik Ł., Zielinska N., Piagkou M., Ananiev J., Dimitrova I.N., Slavchev S.A. (2024). Are There Any Differences in the Healing Capacity between the Medial Collateral Ligament’s (MCL) Proximal and Distal Parts in the Human Knee? Quantitative and Immunohistochemical Analysis of CD34, α-Smooth Muscle Actin (α-SMA), and Vascular Endothelial Growth Factor (VEGF) Expression Regarding the Epiligament (EL) Theory. Biomedicines.

[4] Vosoughi F., Rezaei Dogahe R., Nuri A., Ayati Firoozabadi M., Mortazavi J. (2021). Medial collateral ligament injury of the knee: A review on current concept and management. Arch. Bone Jt. Surg..

[5] DeLong J., Waterman B. (2015). Surgical techniques for the reconstruction of medial collateral ligament and posteromedial corner injuries of the knee: A systematic review. Arthroscopy.

[6] Memarzadeh A., Melton J. (2019). Medial collateral ligament of the knee: Anatomy, management and surgical techniques for reconstruction. Orthop. Trauma.

[7] Yaras R.J., O’Neill N., Mabrouk A., Yaish A.M. (2026). Lateral collateral ligament knee injury. StatPearls [Internet].

[8] Georgiev G.P., Iliev A., Kotov G., Kinov P., Slavchev S., Landzhov B. (2017). Light and electron microscopic study of the medial collateral ligament epiligament tissue in human knees. World J. Orthop..

[9] Petersen W., Tillmann B. (1999). Structure and vascularization of the cruciate ligaments of the human knee joint. Anat. Embryol..

[10] Bjur D., Alfredson H., Forsgren S. (2005). The innervation pattern of the human Achilles tendon: Studies of the normal and tendinosis tendon with markers for general and sensory innervation. Cell Tissue Res..

[11] Gaydarski L., Landzhov B., Tubbs R.S., Georgiev G.P. (2026). Can the Spatial Heterogeneity in the Epiligament Explain the Differential Healing Capacities of the ACL and MCL?. J. Clin. Med..

[12] Woo S.L.Y., Abramowitch S.D., Kilger R., Liang R. (2006). Biomechanics of knee ligaments: Injury, healing, and repair. J. Biomech..

[13] Hol E.M., Capetanaki Y. (2017). Type III intermediate filaments desmin, glial fibrillary acidic protein (GFAP), vimentin, and peripherin. Cold Spring Harb. Perspect. Biol..

[14] Danielsson F., Peterson M.K., Caldeira Araújo H., Lautenschläger F., Gad A.K.B. (2018). Vimentin diversity in health and disease. Cells.

[15] Parvanian S., Coelho-Rato L.S., Eriksson J.E., Patteson A.E. (2023). The molecular biophysics of extracellular vimentin and its role in pathogen–host interactions. Curr. Opin. Cell Biol..

[16] Wang J.H.C. (2006). Mechanobiology of tendon. J. Biomech..

[17] Donato R., Cannon B.R., Sorci G., Riuzzi F., Hsu K., Weber D.J., Geczy C.L. (2013). Functions of S100 proteins. Curr. Mol. Med..

[18] Leśniak W., Filipek A. (2023). S100A6 protein—Expression and function in norm and pathology. Int. J. Mol. Sci..

[19] Bhattacharya S., Bunick C.G., Chazin W.J. (2004). Target selectivity in EF-hand calcium binding proteins. Biochim. Biophys. Acta.

[20] Xia C., Braunstein Z., Toomey A.C., Zhong J., Rao X. (2018). S100 proteins as an important regulator of macrophage inflammation. Front. Immunol..

[21] Kyo Y., Kato K., Park Y.S., Gajhate S., Umehara T., Lillehoj E.P., Suzaki H., Kim K.C. (2012). Antiinflammatory role of MUC1 mucin during infection with nontypeable *Haemophilus influenzae*. Am. J. Respir. Cell Mol. Biol..

[22] Nath S., Mukherjee P. (2014). MUC1: A multifaceted oncoprotein with a key role in cancer progression. Trends Mol. Med..

[23] Chen W., Zhang Z., Zhang S., Zhu P., Ko J.K.S., Yung K.K.L. (2021). MUC1: Structure, function, and clinic application in epithelial cancers. Int. J. Mol. Sci..

[24] Grewal U.S., Kurzrock R. (2025). Mucin-1: A promising pan-cancer therapeutic target. npj Precis. Oncol..

[25] Kumar P., Ji J., Thirkill T.L., Douglas G.C. (2014). MUC1 is expressed by human skin fibroblasts and plays a role in cell adhesion and migration. BioRes. Open Access.

[26] Georgiev G.P., Gaydarski L., Landzhov B. (2025). Should We Accept the Epiligament Theory About the Differences in the Healing Potential of the Medial Collateral and the Anterior Cruciate Ligament?. Biomedicines.

[27] Georgiev G.P., Yordanov Y., Olewnik Ł., Tubbs R.S., LaPrade R.F., Ananiev J., Slavchev S.A., Dimitrova I.N., Gaydarski L., Landzhov B. (2024). Do the Differences in the Epiligament of the Proximal and Distal Parts of the Anterior Cruciate Ligament Explain Their Different Healing Capacities? Quantitative and Immunohistochemical Analysis of CD34 and α-SMA Expression in Relation to the Epiligament Theory. Biomedicines.

[28] Kholinne E., Lee H.J., Kim G.Y., Deslivia M.F., Adikrishna A., Bin Z., Lee S.J., Rhyu I.J., Lim S.J., Hong H.P. (2018). Mechanoreceptors distribution in the human medial collateral ligament of the elbow. Orthop. Traumatol. Surg. Res..

[29] Frank C.B. (2004). Ligament structure, physiology and function. J. Musculoskelet. Neuronal Interact..

[30] Benjamin M., Toumi H., Ralphs J.R., Bydder G., Best T.M., Milz S. (2006). Where tendons and ligaments meet bone: Attachment sites (‘entheses’) in relation to exercise and/or mechanical load. J. Anat..

[31] Miron-Mendoza M., Poole K., DiCesare S., Nakahara E., Bhatt M.P., Hulleman J.D., Petroll W.M. (2024). The Role of Vimentin in Human Corneal Fibroblast Spreading and Myofibroblast Transformation. Cells.

[32] Docheva D., Müller S.A., Majewski M., Evans C.H. (2015). Biologics for tendon repair. Adv. Drug Deliv. Rev..

[33] Sakaguchi M., Miyazaki M., Kondo T., Namba M. (2001). Up-regulation of S100C in normal human fibroblasts in the process of aging in vitro. Exp. Gerontol..

